# Supplementary material for: Research on high performance combustible gas concentration sensor based on pyramid beam splitter matrix
Source: PLoS One. 2024 May 16;19(5):e0300374. doi: 10.1371/journal.pone.0300374 (PMC11098315; doi:10.1371/journal.pone.0300374)
Supplement: Attachment — Submitted filename: PONE-D-23-32114 Reviewer Report.pdf [file pone.0300374.s002.pdf]

Reviewer Report

Overview

This work is focused on using a pyramid beam splitter matrix to improve gas sensor accuracy and sensitivity. The novelty of the work is the use of beam splitter design to demonstrate the impact of increasing light path length to the sensor performance. The topic would be of interest to the readers but requires clarifications and revisions. Hence the recommendation is to publish with minor revision.

Overall Comments:

- Formatting and font is not consistent and should be made consistent.

1 Introduction

The introduction mentions the problem statement targeted and motivation to pursue a system with better accuracy and sensitivity. The following addition will make the introduction more robust:

- Authors should add typical values of sensor accuracy and sensitivity observed to provide readers a reference to compare the current work's results. Appropriate citations should be added for the claimed values.

2 Design of sensitive element and measurement circuit

This section explains the fundamentals of the sensor principle, hardware and circuit well. The following addition will help further improve the quality of the work:

- The authors should mention that the calculations are discussed in the subsequent section to provide context to the readers for the line 114-116.

3 Combustible gas concentration detection algorithm based on CZT principle

This section does a good work in introducing the calculations used to determine the sensitivity and accuracy of the device. The following addition should be made:

- Add citation for line 145-146: "CZT algorithm is often used for spectrum refinement in the characteristic bands of the signal, 146 and it has the advantages of flexible refinement scale and high accuracy."
- The authors mention in line 154-155 "We can derive equation (2) from equation (3)." It seems it should be the other way. Please clarify or modify accordingly.

4 Experiments and results

- Authors should clarify on line 262 "The maximum value of the concentration variance of S^2_{COL} the detection result was 0.014PPM, and the minimum one was 0.0015PPM, indicating that the trusted accuracy of the concentration detection result can reach 0.014PPM level in the most adverse condition." what is meant by most adverse condition here and how has this experiment demonstrated this.

- For figure 11, authors should mention in the caption that the figure also includes data for 1 ppm methane.

- For the table 2 the authors should clarify in the caption that the data is only for 0.5 ppm methane.

- In section 4.2 The authors should comment on the delta between the expected concentration of the methane 5.0 ppm and the obtained values of 5.0380 ppm. The authors should additionally clarify how many times was this data collection performed. (Optional: Adding repeatability and reproducibility experiments would add robustness to the work).

- The authors should clarify why the tested concentrations in section 4.3, 4.4 and 4.5 were specifically 30/70, 20/60 and 40/80 respectively.

- Table 4 column mentions concentration of 20 twice. Correct one of them to 60 appropriately.

- The authors should Clarify why the dust concentration was chosen as 100 mg/m3. Mention how this emulates a real world situation.

5 Conclusion

The conclusion provides a high level summary of the key insights from the work. However the conclusion should be more quantitative. The authors should also clarify if this conclusion can be translated for other types of gases as well since the current work focuses on methane. If so the authors should clarify why.
